# Structural Connectivity of the Anterior Cingulate Cortex, Claustrum, and the Anterior Insula of the Mouse

**DOI:** 10.3389/fnana.2018.00100

**Published:** 2018-11-26

**Authors:** Houman Qadir, Samuel R. Krimmel, Chaoqi Mu, Alexandros Poulopoulos, David A. Seminowicz, Brian N. Mathur

**Affiliations:** ^1^Department of Pharmacology, University of Maryland School of Medicine, Baltimore, MD, United States; ^2^Department of Neural and Pain Sciences, School of Dentistry, Center to Advance Chronic Pain Research, University of Maryland, Baltimore, MD, United States

**Keywords:** retrograde, anterograde, striatum, endopiriform, circuitry, salience network

## Abstract

The claustrum is a narrow subcortical brain structure that resides between the striatum and insular cortex. The function of the claustrum is not fully described, and while our previous work supports a role for the claustrum in top-down cognitive control of action, other evidence suggests the claustrum may be involved in detecting salient changes in the external environment. The anterior cingulate cortex (ACC) and the anterior insular (aINS) are the two major participants in the salience network of human brain regions that activate in response to salient stimuli. While bidirectional connections between the ACC and the claustrum exist from mouse to non-human primate, the aINS connectivity with claustrum remains unclear, particularly in mouse. Here, we explored structural connections of the aINS with the claustrum and ACC through adeno-associated virus neuronal tract tracer injections into the ACC and aINS of the mouse. We detected sparse projections from the claustrum to the aINS and diffuse projections from the aINS to the borders of the claustrum were observed in some cases. In contrast, the insular cortex and endopiriform nucleus surrounding the claustrum had rich interconnectivity with aINS. Additionally, we observed a modest interconnectivity between ACC and the aINS. These data support the idea that claustrum neuron responses to salient stimuli may be driven by the ACC rather than the aINS.

## Introduction

Many cortical areas bidirectionally connect with the claustrum across mammalian species, including primate (Bianchi, 1922; Rae, 1954; Markov et al., 2011; Reser et al., 2014), cat (LeVay and Sherk, 1981; Markowitsch et al., 1984; Witter et al., 1988), rat (White et al., 2017), and mouse (Wang et al., 2017). The claustrum does not equally connect to all cortical areas, however. For instance, the anterior cingulate cortex (ACC) more strongly projects to and receives connections from the claustrum. Conversely, primary somatomotor cortices do not strongly project to and receive projections from the claustrum (White et al., 2017).

A frontal cortical area that would be expected to significantly connect with the claustrum is the anterior insular cortex (aINS), which, along with the claustrum, is implicated in salience detection (Craig, 2002, 2009; Mathur et al., 2009; Menon and Uddin, 2010; Remedios et al., 2010; Mathur, 2014). However, in previous studies that examined aINS connectivity with claustrum, the results are unclear. In the cat, no claustro-insular connection following injections of horseradish peroxidase in the aINS were observed (Markowitsch et al., 1984). In the rat and rabbit, however, connections from the aINS and endopiriform nucleus to the dorsal claustrum region were detected (Lipowska et al., 2000). One reason for these discrepancies may be that the anatomical boundaries of the claustrum in these studies were not defined by data-driven anatomical criteria.

In the mouse, the connection between the aINS and the claustrum is unexplored, while the anatomical boundaries of the claustrum are well-defined (White et al., 2018). As such, this species represents an appropriate model to accurately describe the connections between the claustrum and the aINS. The anatomical boundaries of the rodent claustrum are defined by the claustrum-specific protein marker, Gng2, and the expression of this marker is isomorphic with parvalbumin (PV) immunostaining in rat (Mathur et al., 2009) and mouse (White et al., 2018). The Gng2- and PV-defined claustrum is also consistent with the boundaries of the claustrum as defined by (1) neuronal somata that send projections to the ACC; and (2) axonal terminations arising from the ACC in both the rat (Smith et al., 2012; White et al., 2017) and the mouse (White et al., 2018). In light of this, the mouse also represents an appropriate model to examine the poorly studied rodent structural connection between the ACC and the aINS, which are two structures in human that are part of the so-called salience network (Taylor et al., 2009; Uddin, 2015).

To describe the mouse structural connections between the aINS, ACC, and claustrum, in this study we injected one adeno-associated virus (AAV) expressing a green fluorophore into the ACC and another AAV virus expressing a red fluorophore into the aINS, and vice versa. Both viruses exhibit both anterograde and retrograde trafficking properties (Tervo et al., 2016). While we observed dense labeling of claustrum neurons projecting to the ACC, no claustrum neurons were observed projecting to the aINS. Furthermore, we observed dense projections from aINS to the insular cortex and endopiriform nucleus that surrounds the claustrum and diffuse anterograde labeling within the claustrum. Sparse bidirectional connections between ACC and aINS were also observed. These findings carry implications for the structural basis of the salience network and the contribution of the claustrum to salience encoding.

## Materials and Methods

### Animals

Four C57BL/6J wild-type mice of both sexes were used for this study. Mice were 10–13 weeks of age at the time of surgery and euthanasia. Mice were group-housed with food and water available *ad libitum*, and on a 12-h day/night cycle beginning at 07:00 each day. This study was reviewed and approved by the National Institutes of Health Guide for Care and Use of Laboratory Animals and the University of Maryland, School of Medicine Animal Care and Use Committee.

### Stereotaxic Procedures and Viral Vectors

Mice were anesthetized via inhalation of 3.5% isoflurane and placed in a mouse stereotaxic frame while anesthesia was maintained with inhalation of 1% isoflurane. A stereotaxic drill was used to drill small openings in the mouse skull above brain regions prior to viral injection. Viral injections in the ACC and aINS were performed with either 100 nL of retrograde AAV vector expressing a green fluorescent protein under the *hSyn* (human synapsin) promoter (rAAV2-retro-JAWS-KGC-GFP; Addgene reference #65014-AAVrg; Plasmid #81070) or 100 nL of retrograde AAV expressing a td Tomato tag under the *CAG* (chicken beta-actin) promoter (rAAV2-retro-CAG-td-tomato; Addgene reference #59462-AAVrg; Plasmid #81070) (Tervo et al., 2016). We controlled for any fluorescence and expression time differences among the two viruses by counterbalancing them in experimental animals. Injections in both regions were unilateral and performed either ipsilaterally or contralaterally. Relative to bregma, the coordinates used for ACC injections were anterior-posterior (AP): +1.0 mm, medial-lateral (ML): ±0.3 mm, dorsal-ventral (DV): -1.1 mm. The coordinates used for aINS were AP: +1.94 mm, ML: ±2.5 mm, DV: -3.5 mm. DV coordinates were measured from top of brain surface.

### Histology

Mice were overdosed on isoflurane and perfused with room temperature 0.1 M phosphate-buffered solution (PBS), pH 7.3, and then with ice-cold 4% paraformaldehyde (PFA) solution in PBS, 10 days after viral injection surgery. After extraction, the brains were post-fixed in 4% PFA solution overnight. Fifty micrometer thickness slices were obtained using the Integraslice 7550 MM vibrating microtome (Campden Instruments, Loughborough, England) and were stored at 4°C in 0.1 M PBS. The slices were mounted onto 25 × 75 × 1 mm frosted microscope slides (Thermo-Scientific, Waltham, MA, United States) using 127 μL of ProLong Gold antifade reagent (Invitrogen, Carlsbad, CA, United States) as the mountant. The slides were imaged using a Nikon fluorescence microscope (Nikon, Minato, Tokyo, Japan) with images obtained using both 4× and 10× magnification objectives. 10× images of slice sections were obtained and stitched together with 10% blending to create a complete field of view image of the entire brain slice. Adobe Illustrator was used to create chartings depicting viral tracer deposits.

## Results

**FIGURE 1:**
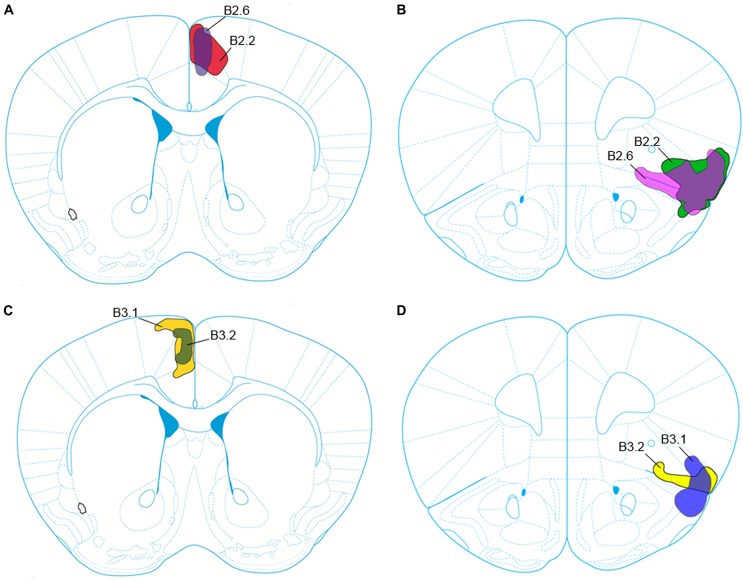
Schematic representation of the core and penumbra of the viral tract tracer injection into the anterior cingulate cortex (ACC) for cases B2.2 and B2.6 **(A)**, and into the anterior insular cortex (aINS) for cases B2.2 and B2.6 **(B)**. Viral tract tracer injection core and penumbra for cases B3.1 and B3.2 into the ACC are depicted in **(C)** and into the aINS depicted in **(D)**.

We followed the nomenclature of Paxinos and Watson (2014) for the regions of interest at various rostrocaudal levels. The four cases (B2.2, B2.6, B3.1, and B3.2) presented include labeled cells after viral tract tracer deposits injected into the ACC and anterior insula. The viruses used expressed either a green fluorophore (rAAV2-retro-JAWS-KGC-GFP) or a red fluorophore (rAAV2-retro-CAG-td-tomato). These viruses were used for their high sensitivity owing to efficient retrograde and anterograde trafficking and ability to continually express fluorophore. We distinguished retrograde cells from anterograde labeling by observing cell body and dendrite labeling. Anterograde labeling was determined by axon termini expressing fluorophore that presented as weak background fluorescence. It is important to note that we are unable to clearly distinguish between secondary anterograde labeling from retrogradely labeled cell bodies and dendrites. Viruses and injection sites were counterbalanced to control for potential differences in the ability of the promoters to express fluorophore. However, we found no apparent differences in fluorophore expression between viruses (Supplementary Figure S1). Both anterograde and retrograde labeling are described below for each case. A schematic representation of all viral tracer deposits is shown in Figure 1. For all cases, retrogradely labeled neurons were abundant in the ipsilateral claustrum following virus injection into the ACC, as previously reported in mouse (White and Mathur, 2018). This labeling is isomorphic with Gng2- and PV-immunostaining and was used as the definition of claustrum boundaries in this study.

### Case B2.2

#### ACC Injection Site

**FIGURE 2:**
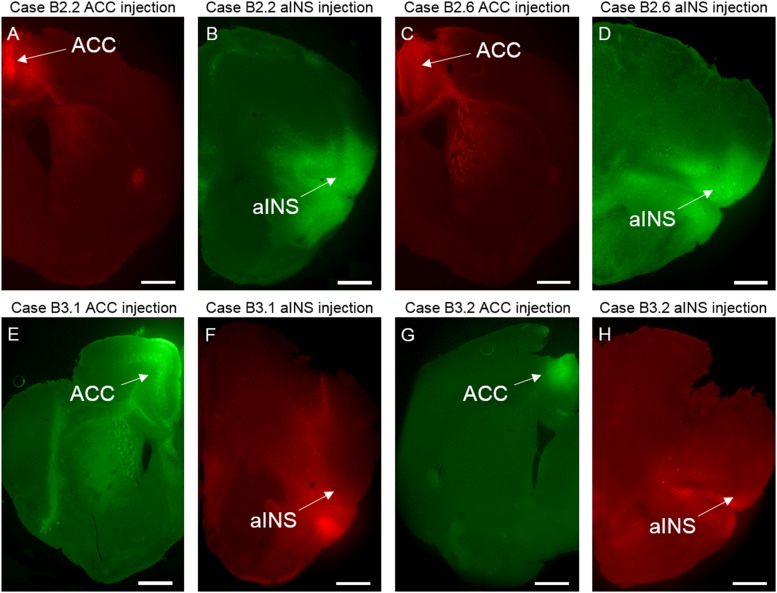
Photomicrographs of viral tracer injections for case B2.2 in ACC **(A)** and aINS **(B)**, for case B2.6 in ACC **(C)** and aINS **(D)**, for case B3.1 in ACC **(E)** and aINS **(F)**, and for case 3.2 in ACC **(G)** and aINS **(H)**. Scale bar: 1 mm.

The core of the first viral tracer (rAAV2-retro-CAG-td-tomato) injection site (Figure 2A) spanned superficial and deep layers of the dorsal and ventral ACC. The penumbra of the injection site spread slightly into the laterally lying secondary motor cortex (M2). The extent of all viral deposition was unilateral in nature. Rostrocaudally, the core of the injection site spanned from AP = +1.10 mm to +0.14 mm relative to bregma and did not extend beyond the rostrocaudal boundaries of the ACC.

#### Retrograde Labeling Following Injection Into the ACC

**FIGURE 3:**
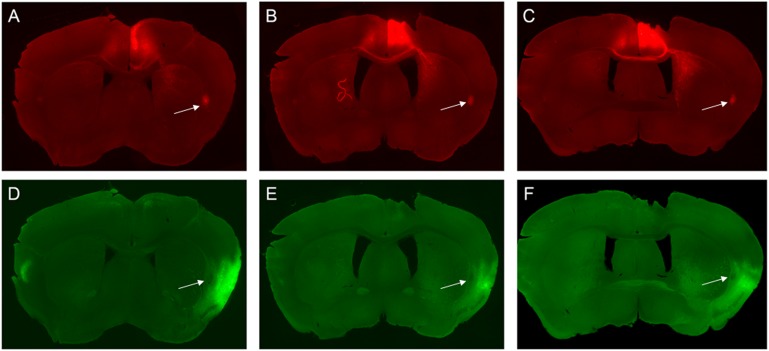
Photomicrographs of the claustrum and surrounding regions at three different rostrocaudal levels of claustrum (CL). Case B2.2 AP = +1.10 mm to +0.14 mm relative to bregma shown. The distribution of labeled elements on the ipsilateral side of viral injection in ACC is shown at rostral **(A)** to mid (**B**) to caudal levels **(C**). The distribution of labeled elements resulting from viral tracer injection into the aINS is shown in the same sections **(D–F)**. Arrows point to claustrum.

**FIGURE 4:**
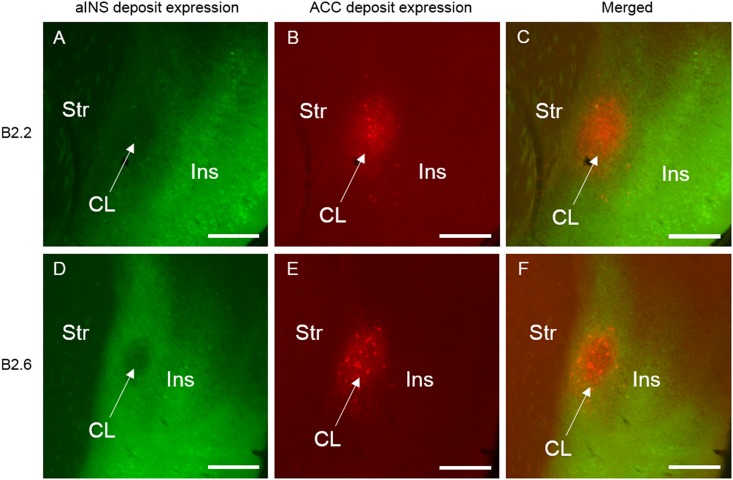
Selected photomicrographs of the rostral level of the claustrum and surrounding regions illustrating retrograde and anterograde labeling following aINS and ACC viral injections (AP = +1.10 mm from bregma) for case B2.2 **(A–C)** and B2.6 **(D–F)**. Dense retrograde and anterograde labeling in the insular cortex, endopiriform nucleus, and piriform areas is shown following viral injection in aINS for both cases **(A,D)**. Retrograde and anterograde labeling of the claustrum following deposit into the ACC **(B,E)**. Merge of panels **(A)** and **(B)** is shown in **(C)**. Merge of **(D)** and **(E)** is shown in **(F)**. Scale bar: 500 μm. Str, striatum; CL, claustrum; Ins, insular cortex.

**FIGURE 5:**
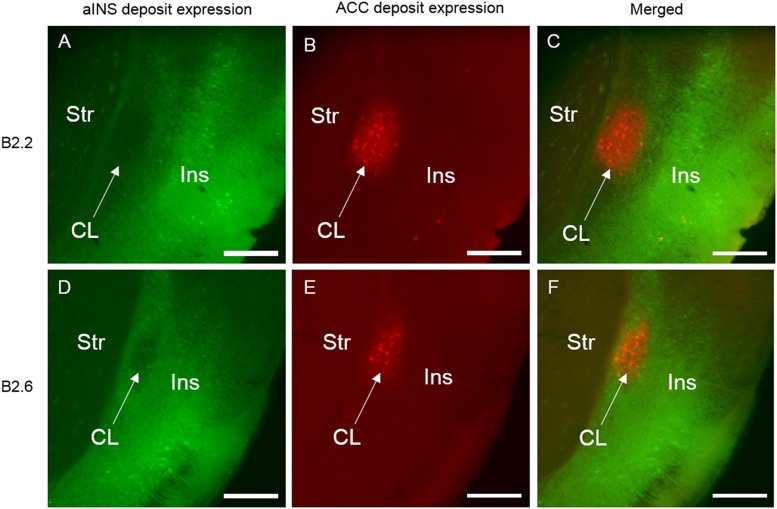
Selected photomicrographs of the mid level of the claustrum and surrounding regions for case B2.2 **(A–C)** and B2.6 **(D–F)** (AP = +0.50 mm from bregma), illustrating retrograde and anterograde labeling following aINS and ACC viral injections including surrounding regions. Merge of panels **(A)** and **(B)** is shown in **(C)**. Merge of **(D)** and **(E)** is shown in **(F)**. Scale bar: 500 μm. Str, striatum; CL, claustrum; Ins, insular cortex.

**FIGURE 6:**
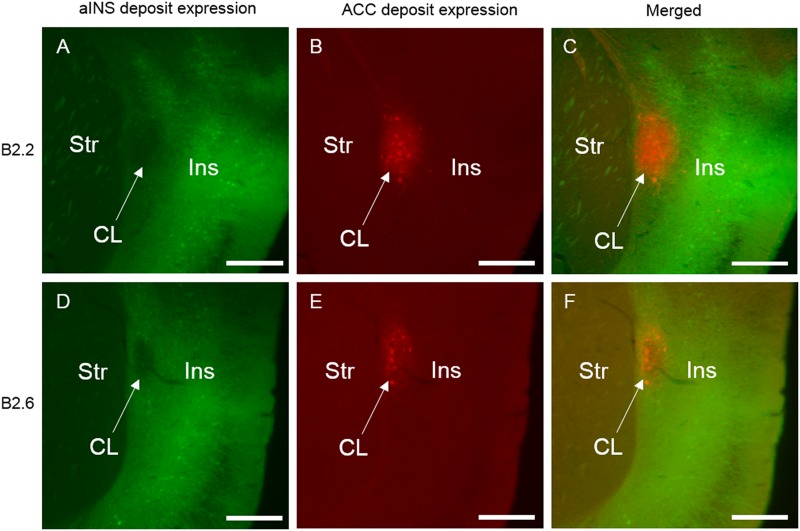
Selected photomicrographs of the caudal level of the claustrum and surrounding regions for case B2.2 **(A–C)** and B2.6 **(D–F)** (AP = +0.14 mm from bregma), illustrating retrograde and anterograde labeling following aINS and ACC viral injections. Merge of panels **(A)** and **(B)** is shown in **(C)**. Merge of **(D)** and **(E)** is shown in **(F)**. Scale bar: 500 μm. Str, striatum; CL, claustrum; Ins, insular cortex.

**FIGURE 7:**
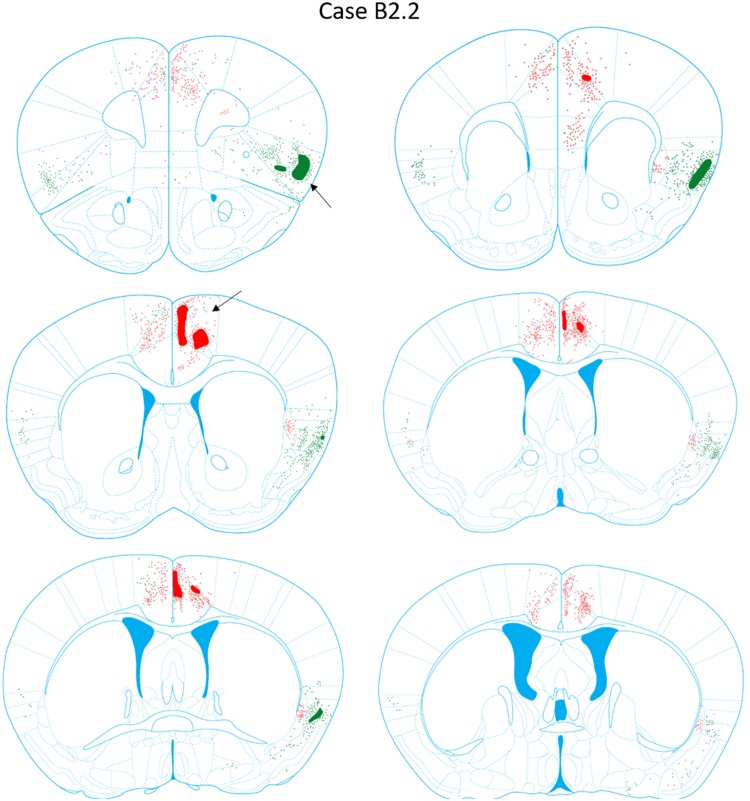
Chartings of retrogradely labeled cells following viral injections into the aINS (green) and ACC (red) (case B2.2). The injection site cores are indicated by black arrows. The chartings were made onto plates modified from Paxinos and Watson (2014).

Labeling of claustrum neurons projecting to ACC spanned throughout the rostrocaudal and dorsoventral axes of the claustrum from AP = +1.54 to -0.58 mm (Figures 3A–C). Retrograde labeling was also observed in more caudal aspects of the cingulate cortex (AP level +0.14 to -0.46 mm). Moderate labeling was apparent in the infralimbic cortex (IL) in sections rostral to the ACC injection site (AP level +1.70 to +1.34 mm). Retrograde labeling was detected in a sparse population of neurons in the deep layers of insular cortex neighboring the claustrum (Figures 4–6) and in the dorsal aspect of piriform area 2 (Pir2). Very few labeled cells were observed in the aINS. Only a few cells labeled in the claustrum contralateral to the viral injection side were found (Figure 7).

#### Anterograde Labeling Following Injection Into the ACC

**FIGURE 8:**
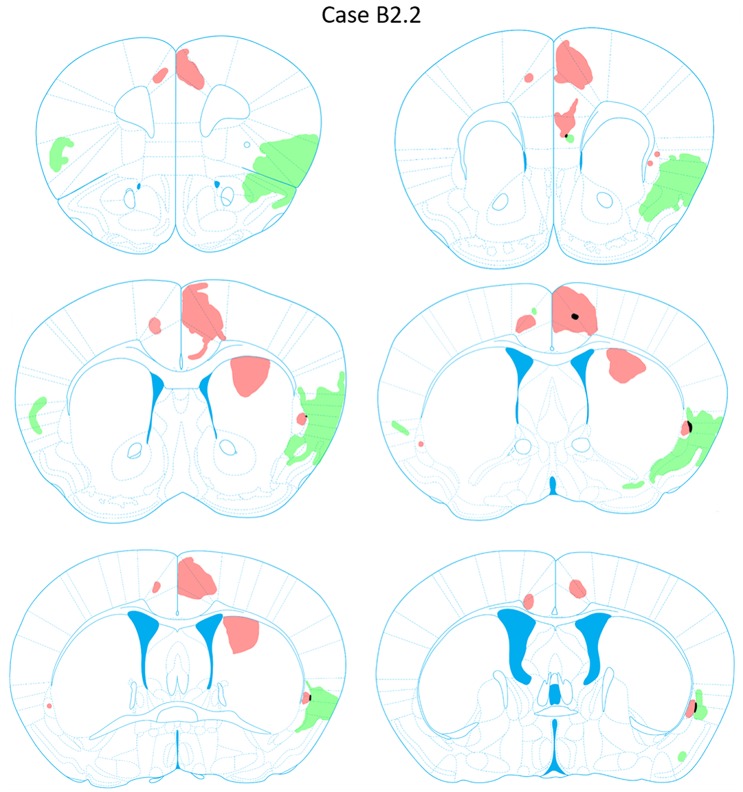
Chartings of anterograde labeling following viral injections into the aINS (green) and ACC (red) (case B2.2). Overlap in expression is colored in black.

Anterograde labeling was also detected following viral injections (Tervo et al., 2016). Anterogradely-labeled axonal fibers were abundant in other areas of ACC and ipsilateral claustrum at nearly all rostrocaudal levels (Figure 8). Anterograde labeling was greatest in the caudal aspect of the claustrum. To a lesser degree, labeling was observed in the contralateral claustrum at AP level +0.38 to +0.14 mm. Anterogradely labeled terminals were dispersed throughout the entire dorsoventral aspect of the claustrum when present. Labeled terminals were also abundant in the dorsomedial striatum (AP = +1.10 to +0.14 mm) and labeled white matter bundles were also observed throughout the dorsal striatum in the same rostrocaudal levels. Labeled fibers were seen in superficial layers of the aINS. Very weak labeling was observed in the deeper layers of the agranular insular cortex adjacent to the caudal aspect of the claustrum (AP = +0.14 to -0.10 mm) but no labeling was apparent in the superficial layers of the agranular insular cortex. Other regions with anterograde labeling detected were the deep layers of the prelimbic cortex (PrL) and the superficial layers of the IL.

#### aINS Injection Site

The core of the aINS viral tracer (rAAV2-retro-JAWS-KGC-GFP) injection for B2.2 (Figure 2B) was in the superficial and deep layers of the aINS. The core of the injection spread spanned from AP = +1.98 to +1.54 mm relative to bregma (Figure 7). The penumbra of the injection spread into the piriform area 2 (Pir2), secondary somatosensory cortex (S2), and the lateral orbital cortex (LO).

#### Retrograde Labeling Following Injection Into the aINS

For the aINS injection, retrograde labeling was primarily concentrated in the superficial layers of the granular and agranular insular cortex (Figures 4–6). The majority of cell labeling was observed ipsilateral to the aINS injection site. Labeling of insular cortex neurons projecting to the aINS spanned throughout the insular cortex rostrocaudal axis from roughly AP = +1.98 mm to -1.06 mm (Figures 3D–F). Caudal sections (AP = +0.14 to -0.10 mm) showed denser retrograde labeling in deeper layers of the insular cortex and immediately adjacent to claustrum but not within the claustrum itself, as defined by retrograde labeling arising from the injection within the ACC (Figures 4–6). A few retrogradely labeled cells were observed in the superficial layers bilaterally of both M2 and the dorsal region of ACC. In addition, moderate retrograde labeling was detected in piriform area 1 (Pir1), dorsal endopiriform (DEn), and intermediate endopiriform (IEn) (AP level +1.34 to +0.14 mm).

#### Anterograde Labeling Following Injection Into the aINS

Following aINS viral injection, dense anterograde labeling of axonal fibers were found throughout all layers of the granular and agranular insular cortex at all rostrocaudal levels (AP = +1.54 to -0.46 mm). Sparse anterograde labeling was also found in the granular and agranular insular cortex on the contralateral side of the aINS injection site. Moderate anterograde labeling of axonal fibers were found in the piriform areas and the endopiriform nucleus between AP levels +1.54 and +0.38 mm. Sparse anterograde labeling of axonal terminals was detected in the superficial layers of the ACC from AP = +0.62 to +0.50 mm. Lastly, there was sparse innervation of the border region of the claustrum on the ipsilateral side of injection site in select sections (AP level +0.98 to +0.14 mm).

### Case B2.6

#### ACC Injection Site

The viral tract tracer (rAAV2-retro-CAG-td-tomato) injection site core spanned all layers of the ventral and dorsal ACC and extended rostrocaudally from AP = +1.18 to +0.38 mm relative to bregma (Figure 2C). The penumbra of the injection site spread to the superficial layers of secondary motor cortex (M2).

#### Retrograde Labeling Following Injection Into the ACC

**FIGURE 9:**
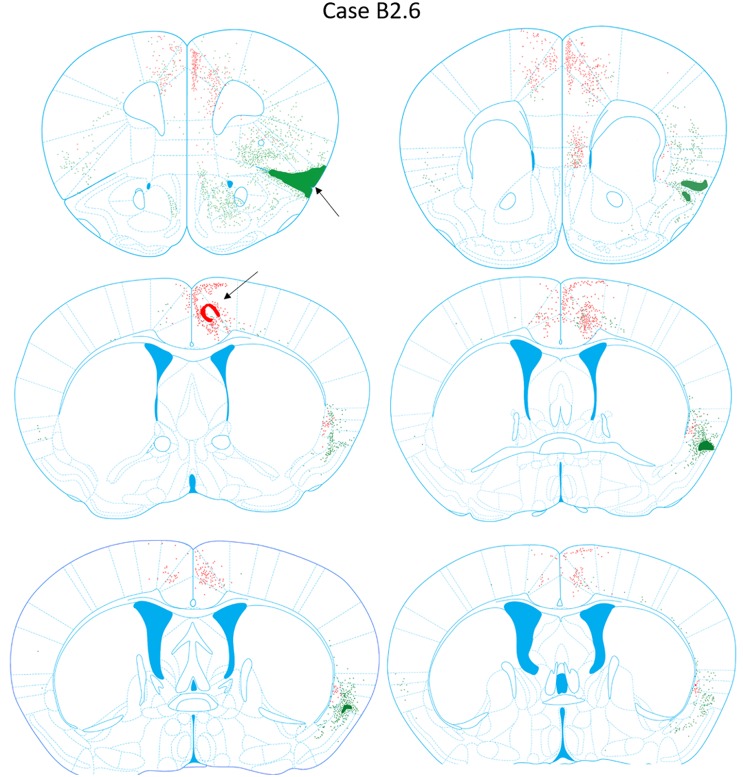
Chartings of retrogradely labeled cells following viral injections into the aINS (green) and ACC (red) (case B2.6). The injection site cores are indicated by black arrows.

Retrogradely labeled neurons in the claustrum along the entire rostrocaudal and dorsoventral axes were present ipsilateral to the injection site from AP = +1.54 to -1.06 mm (Figure 9). Rostral and caudal portions of ACC (AP = +1.98 and +0.14 mm) also contained retrogradely labeled cells. Sparse retrogradely labeled neurons were also present in mostly the deep layers of aINS. Other structures with moderate labeling included the IL and dorsal peduncular area (DP) in sections rostral to the ACC injection site (AP +1.70 to +1.54 mm).

#### Anterograde Labeling Following Injection Into the ACC

**FIGURE 10:**
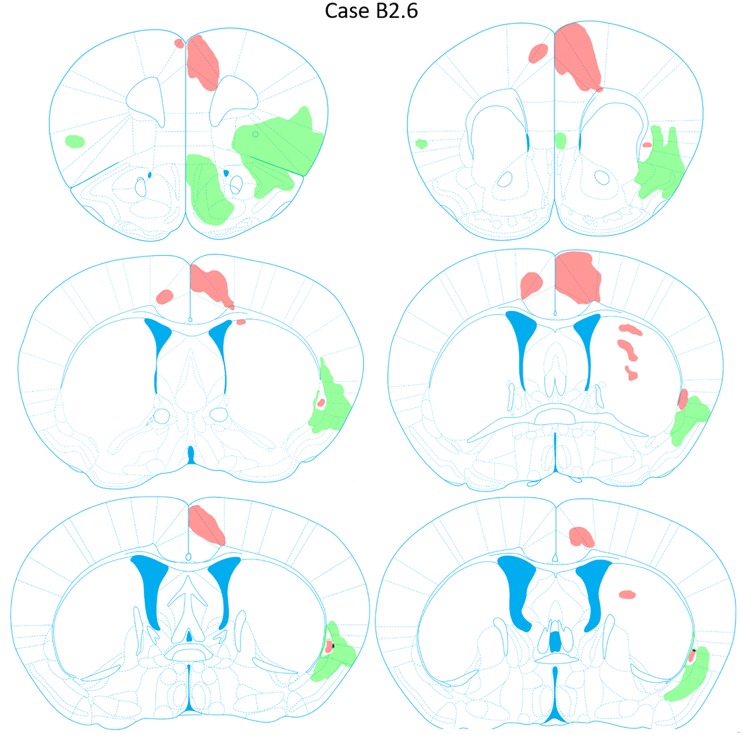
Chartings of anterograde labeling following viral injections into the aINS (green) and ACC (red) (case B2.6). Overlap in expression is colored in black.

Anterogradely labeled axonal fibers were detected at all rostrocaudal levels of the ACC (AP level +1.98 to +0.02 mm) following ACC viral injection (Figure 10). Anterograde labeling in caudal ACC sections (AP = -0.10 to -0.48 mm) was observed in the deeper layers of the ACC. Labeled terminals were observed in the claustrum bilaterally. Such terminals in the claustrum were found to be denser in caudal claustrum sections compared to rostral claustrum sections (AP = +0.14 to 0.10 mm). Labeled terminals were also found in portions of the dorsomedial striatum from AP = +0.26 to +0.14 mm relative to bregma. No labeling was detected in the aINS or agranular and granular insular cortex regions. Lastly, labeling in the ACC contralateral to the ACC injection site was detected (AP = +1.54 to +0.14 mm).

#### aINS Injection Site

The viral deposit (rAAV2-retro-JAWS-KGC-GFP) core was centered primarily in the superficial deep layers of the aINS with parts into the deep layers of aINS and dorsal Pir1 (Figures 2D, 9). The penumbra extended into the S2 superficial layers. Rostrocaudally, the core of the aINS injection site spanned from AP = +2.10 to +1.54 mm relative to bregma. Slight viral spread was also apparent in Pir2.

#### Retrograde Labeling Following Injection Into the aINS

Robust retrograde labeling from the aINS injection was observed in the deep layers of the agranular insular cortex (Figure 9). This labeling was present in the majority of the rostrocaudal axis of the agranular insular cortex (AP = +1.54 to -0.34 mm). Moderate labeling of the ventral aspect of the IL and dorsal aspect of Pir2 was observed (AP = +1.68 to +1.42 mm). In caudal insular cortex sections, retrograde labeling was more prominent in the deep layers of the insular cortex proximal to the claustrum. Minor labeling was observed in the DEn and ventral aspect of S2 (AP = +0.38 to -0.10 mm). Based on the claustrum boundaries formed by retrograde-labeling arising from the ACC injection, few, if any, cells retrogadely labeled from the aINS injection were found within the claustrum labeled region (AP = +0.38 to +0.26 mm). Instead, cells labeled by the aINS injection appeared to surround the claustrum (Figure 9). Lastly, sparse retrograde labeling in the deep layers of ACC was detected throughout the rostrocaudal axis of ACC.

#### Anterograde Labeling Following Injection Into the aINS

Dense anterogradely labeled axonal fibers were observed in the DP, dorsal tenia tecta 1 and 2, the ventral tenia tecta, the anterior olfactory (medial and posterior) area (Figure 10). Anterograde labeling in these regions was found between AP levels +2.22 and +1.70 mm. Labeling was observed throughout the rostrocaudal axes of the aINS and agranular insular cortex (AP = +2.22 to -0.18 mm). Very few terminals were anterogradely labeled within the claustrum boundary. The preponderance of labeled fibers were localized in the area directly surrounding the claustrum in insular cortex. Moderate labeling was observed in the DEn, dorsal portion of IEn, and deep layers of S2 (AP = +1.54 to -0.10 mm). Labeling was present in the contralateral aINS that mirrored the injection site deposit core. However, the density of labeled fibers was much less than the ipsilateral counterpart. Lastly, no anterograde labeling was evident in the ACC.

### Other Cases

For cases B3.1 and B3.2 viral injections into the ACC and aINS were reversed with different fluorophores compared to cases B2.2 and B2.6. Virus expressing GFP (rAAV2-retro-JAWS-KGC-GFP) was injected in the ACC and virus expressing td Tomato (rAAV2-retro-CAG-td-tomato) was injected in the aINS. In addition, the ACC injection sites for these cases were on the contralateral side from the injection site in the aINS (Figures 2E,G) in order to account for any interhemispheric differences.

#### ACC Injection Sites

In both cases, the core of the ACC injection sites was in the deep layers of the ACC spanning from AP = +1.10 to +0.90 mm relative to bregma. In case B3.1, the ACC injection spread into the neighboring M2. In case B3.2, the penumbra from the ACC injection weakly spread into the superficial layers of M2.

#### Retrograde Labeling Following Injection Into the ACC

ACC viral injections resulted in retrograde labeling of claustrum neurons spanning from AP = +1.54 to -0.46 mm for case B3.1 and AP = +1.10 to +0.02 mm for case B3.2. Retrograde labeling was also observed contralaterally and ipsilaterally in the ACC relative to the injection site. For both cases, labeling was more prominent in caudal aspects of cingulate cortex in the deep layers (AP = +0.14 to -0.46 mm). Labeling was also observed in both superficial layers of the IL and DP regions in both cases. No labeling was found in the insular cortex in the rostral aspect of the claustrum for both cases (Supplementary Figure S2). However, sparse labeling was found in the deep layers of agranular insular cortex in the mid (Supplementary Figure S3) and caudal aspect (Supplementary Figure S4) of the claustrum for case B3.2 but not in case B3.1. Lastly, no retrogradely labeled cells were seen in the aINS.

#### Anterograde Labeling Following Injection Into the ACC

As with cases B2.2 and B2.6, cases B3.1 and B3.2 displayed abundant labeled axonal fibers in the ACC, dorsomedial striatum, and the claustrum (AP = +1.54 to +0.14 mm). In case B3.2, anterograde fibers were also observed in the deep layers of the IL, but this was not apparent in case B3.1. Weaker anterograde labeling was found in the claustrum on the contralateral side to the ACC injection site. No labeled fibers were detected in the aINS in either case. No fibers were observed in the rostral aspect of the claustrum in case B3.2 and throughout the levels of the claustrum in case B3.1. Sparse fibers appeared at the boundary between claustrum and the deep layers of agranular insular cortex in the mid (Supplementary Figure S3) and caudal levels (Supplementary Figure S4) of the claustrum for case B3.2.

#### aINS Injection Sites

For case B3.1 the core of the aINS injection site was centered in the Pir1 and Pir2 and the penumbra extended into all layers of the aINS (Figure 2F). The center of the aINS injection for case B3.2 was primarily centered in the superficial layers of the aINS and the penumbra spread into the deep layers of the aINS, Pir 1 and 2, and LO (Figure 2H).

#### Retrograde Labeling Following Injection Into the aINS

On the ipsilateral side of the aINS injection retrograde labeling was observed in the granular insular cortex, agranular insular cortex, DEn, and the Pir areas (AP = +1.42 to -0.46 mm). Retrograde labeling density was relatively lower on the contralateral sides from injection sites. No retrograde labeling was detected within the claustrum boundary in the rostral level of the claustrum in case B3.1 and B3.2 (Supplementary Figure S2) and in the mid aspect of case B3.1 (Supplementary Figure S3). Sparse retrograde labeling was observed in the claustrum in the caudal level of the claustrum in case B3.1 (Supplementary Figure S4) and throughout the mid and caudal levels in case B3.2 (AP = +0.60 to +0.10 mm) (Supplementary Figures S3, S4). Lastly, a few labeled neurons were evident in the deep layers of (largely dorsal) ACC for both cases.

#### Anterograde Labeling Following Injection Into the aINS

Following aINS injection in case B3.1 and B3.2, anterogradely labeled axonal fibers were observed throughout the granular and agranular insular cortex (AP = +1.98 to -0.38 mm). Moderate labeling of axonal terminals was present in the Pir areas, superficial layers of the IL, and the deep layers of S2. Both cases revealed anterogradely labeled fibers around, but not within, the claustrum boundary as defined by retrograde labeling arising from the ACC injection site. This was observed throughout the rostrocaudal axis of the claustrum in case B3.1 and 3.2 (Supplementary Figures S2–S4). No labeled fibers were evident in ACC.

## Discussion

The present results indicate that the mouse claustrum weakly, if at all, projects to the aINS. Little input arises from the aINS to the borders of claustrum, and perhaps no input to its core. In contrast, the endopiriform nucleus is bidirectionally connected with the aINS. The labeling of the claustrum and other areas via viral injection into the ACC was in accordance with previous findings in rats (Smith and Alloway, 2010; White et al., 2017) and mouse (White et al., 2018). Finally, a few cells were found to project to the ACC from the aINS, and vice versa.

It is important to consider that the viruses used for this study are different viral constructs that may lead to differences in expression and visualization of neuronal elements. For example, the JAWS viral construct contains a membrane-bound red-shifted halorhodopsin (Chuong et al., 2014), while the td-tomato-expressing virus uses a classical intracellular expression system. However, no differences in fluorescence were detected under high magnification confocal imaging of these labeled neurons (Supplementary Figure S1). For complete control of any confounds, we used both virus types for each injection site targeted. This counterbalancing of viruses also controls for any expression time differences that may arise in using two different viral constructs for tract-tracing. The similar results obtained from all four cases suggest that a difference in virus type does not introduce any notable problems when analyzing the structural connectivity between brain regions, at least in this study.

The differential connectivity of aINS with claustrum and endopiriform nucleus raises the possibility that this variability may be due to differences in aINS injection locations. However, our injections spanned aINS and spread of the injection sites were observed in cortical areas surrounding aINS, such as LO and piriform areas. Given that we observed weak connectivity with claustrum in all the injections targeting aINS, we conclude that these areas may only weakly structurally connect with claustrum as well. The differential connectivity of claustrum and endopiriform nucleus with aINS are interesting considering that the claustrum and endopiriform nucleus are both formed in the deep part of the claustrum primordium in the lateral pallium (Watson and Puelles, 2016). In light of this, our results suggest that the shared developmental origins of the claustrum and endopiriform nucleus may not dictate the final connectivity of these structures with the aINS.

In human imaging studies, the dorsal ACC (dACC) responds to relevant environmental stimuli (Seeley et al., 2007; Uddin, 2015). The anterior insula (aINS) co-activates along with the dACC to generate, along with other structures, the so-called salience network of brain regions (Taylor et al., 2009; Cai et al., 2014; Uddin, 2015; Ellard et al., 2018). In the rhesus monkey, the ACC and aINS are connected only by large Von Economo neurons (VENs) found in both dACC and aINS (Mesulam and Mufson, 1982). VENs are sparsely populated in select primates (Allman et al., 2010) and humans (von Economo and Koskinas, 1925) but are not found in rodents (Hakeem et al., 2009). As such, it is interesting that we observed a bidirectional connection between ACC and aINS. We assume that our ability to detect the ACC-aINS connection in mouse is a result of the high retrograde tracing sensitivity of the viruses used.

Although drawing comparative conclusions between rodent and primate aINS is challenging, and the present results should be interpreted accordingly, there are similarities between the aINS of the rodent and the non-human primate. For example, the granular, dysgranular, and agranular subdivisions of the aINS are conserved across species (Gu et al., 2013; Gogolla, 2017). In addition, the aINS in both species type consists of excitatory pyramidal cells (Anderson et al., 2009; Yamamoto et al., 2010) and GABAergic interneurons (Ohara et al., 2003; Gallay et al., 2012; Gogolla et al., 2014). Furthermore, the aINS in both rodents and primates forms reciprocal connections with cortical areas including auditory, somatosensory, olfactory, gustatory, and visual cortical areas (Aggleton et al., 1980; Augustine, 1996; Zingg et al., 2014). Connections with the lateral and basolateral amygdala in both species types are also evident (Aggleton et al., 1980). However, the circuitry of aINS differs between rodents and primates. In the primate, the aINS reciprocally connects with frontal brain regions such as the ACC, orbitofrontal, and medial prefrontal cortices (Aggleton et al., 1980). Evidence for these connections in the extant rodent literature are inconclusive (Jasmin et al., 2004; Jones et al., 2005). Alternatively, in light of our findings of connectivity between the aINS and the ACC, such differences may be a result of the limited sensitivity of tract tracers used previously.

In humans, multiple functional roles for the aINS are proposed, including an implication in awareness through a close integration with the dACC (Craig, 2009). Another postulated role for the aINS is the integration of sensory stimuli and facilitating the detection of relevant environmental stimuli as part of the so-called “salience network” (Menon and Uddin, 2010). Within the salience network, the aINS is suggested to activate the dACC when salient stimuli are detected (Downar et al., 2000). Indeed, the dACC is activated transiently after changing salient stimuli and the presentation of goal-directed salient stimuli (Han et al., 2018). As the present findings show a poor connection between claustrum and the aINS of mouse, it is possible an alternative brain region is structurally connected to both aINS and ACC/dACC that allows for aINS-dACC co-activation in response to salient stimuli. Our results indicate a common overlap in viral tract tracer expression in the IL. These results are consistent with tract tracing studies conducted in rat that demonstrate structural connections between the aINS and IL (Allen et al., 1991), and ACC and IL (Hurley et al., 1991).

Future analyses of the connections between claustrum, aINS, and the ACC would benefit from being quantified. An anatomical quantification analysis that could be applied is exemplified in a study by Markov et al. (2011); quantification would allow for a determination of statistical significance in connectivity. Ultimately, it is essential to functionally analyze these connections to determine their respective contributions to network formation and behavior. Regardless, assuming that the rodent ACC and aINS are reasonably analogous structures to primate dACC and aINS, and considering the data supporting claustrum detection of salient signals (Remedios et al., 2010, 2014), a reevaluation of the possible role of the claustrum in synchronizing the ACC and aINS with a common glutamatergic input in response to the presentation of a salient stimulus to the organism is warranted. In the least, on hodological grounds the present data support a functional distinction between the insular cortex and the claustrum in the mouse despite their shared association with salience encoding in primate.

## Author Contributions

HQ and BM designed the research. HQ generated the data. HQ, AP, SK, DS, and BM analyzed the data. HQ, SK, DS, CM, and BM wrote the manuscript.

## Conflict of Interest Statement

The authors declare that the research was conducted in the absence of any commercial or financial relationships that could be construed as a potential conflict of interest.

## Supplementary Material

Figure S1 Representative photomicrograph showing viral retrograde expression of rAAV2-retro-JAWS-KGC-GFP **(A)** and rAAV2-retro-CAG-td-tomato **(B)** Scale bars: 50 μm.

Figure S2 Photomicrographs of the rostral level of the claustrum and surrounding regions for case B3.1 **(A–C)** and B3.2 **(D–F)** (AP = +1.10 mm from bregma) showing retrograde and anterograde labeling following viral injections targeting aINS and ACC. Merge of panels **(A)** and **(B)** is shown in **(C)**. Merge of **(D)** and **(E)** is shown in **(F)**. Scale bars: 500 μm. Str, striatum; CL, claustrum; Ins, insular cortex.

Figure S3 Photomicrographs of the mid level of the claustrum and surrounding regions for case B3.1 **(A–C)** and B3.2 **(D–F)** (AP = +0.50 mm from bregma) showing retrograde and anterograde labeling following viral injections targeting aINS and ACC. Merge of panels **(A)** and **(B)** is shown in **(C)**. Merge of **(D)** and **(E)** is shown in **(F)**. Scale bars: 500 μm. Str, striatum; CL, claustrum; Ins, insular cortex.

Figure S4 Photomicrographs of the caudal level of the claustrum and surrounding regions for case B3.1 **(A–C)** and B3.2 **(D–F)** (AP = +0.14 mm from bregma) showing retrograde and anterograde labeling following viral injections targeting aINS and ACC. Merge of panels **(A)** and **(B)** is shown in **(C)**. Merge of **(D)** and **(E)** is shown in **(F)**. Scale bars: 500 μm. Str, striatum; CL, claustrum; Ins, insular cortex.
