# Supplementary figures and images for: Structural Connectivity of the Anterior Cingulate Cortex, Claustrum, and the Anterior Insula of the Mouse
Source: Front Neuroanat. 2018 Nov 26;12:100. doi: 10.3389/fnana.2018.00100 (PMC6276828; doi:10.3389/fnana.2018.00100)

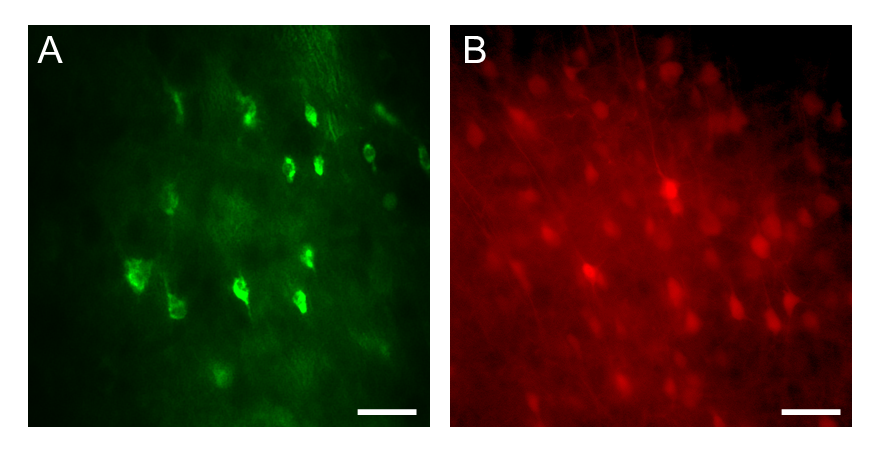

Supplement: Figure S1 — Representative photomicrograph showing viral retrograde expression of rAAV2-retro-JAWS-KGC-GFP (A) and rAAV2-retro-CAG-td-tomato (B) Scale bars: 50 μm. [file Image_1.TIF]

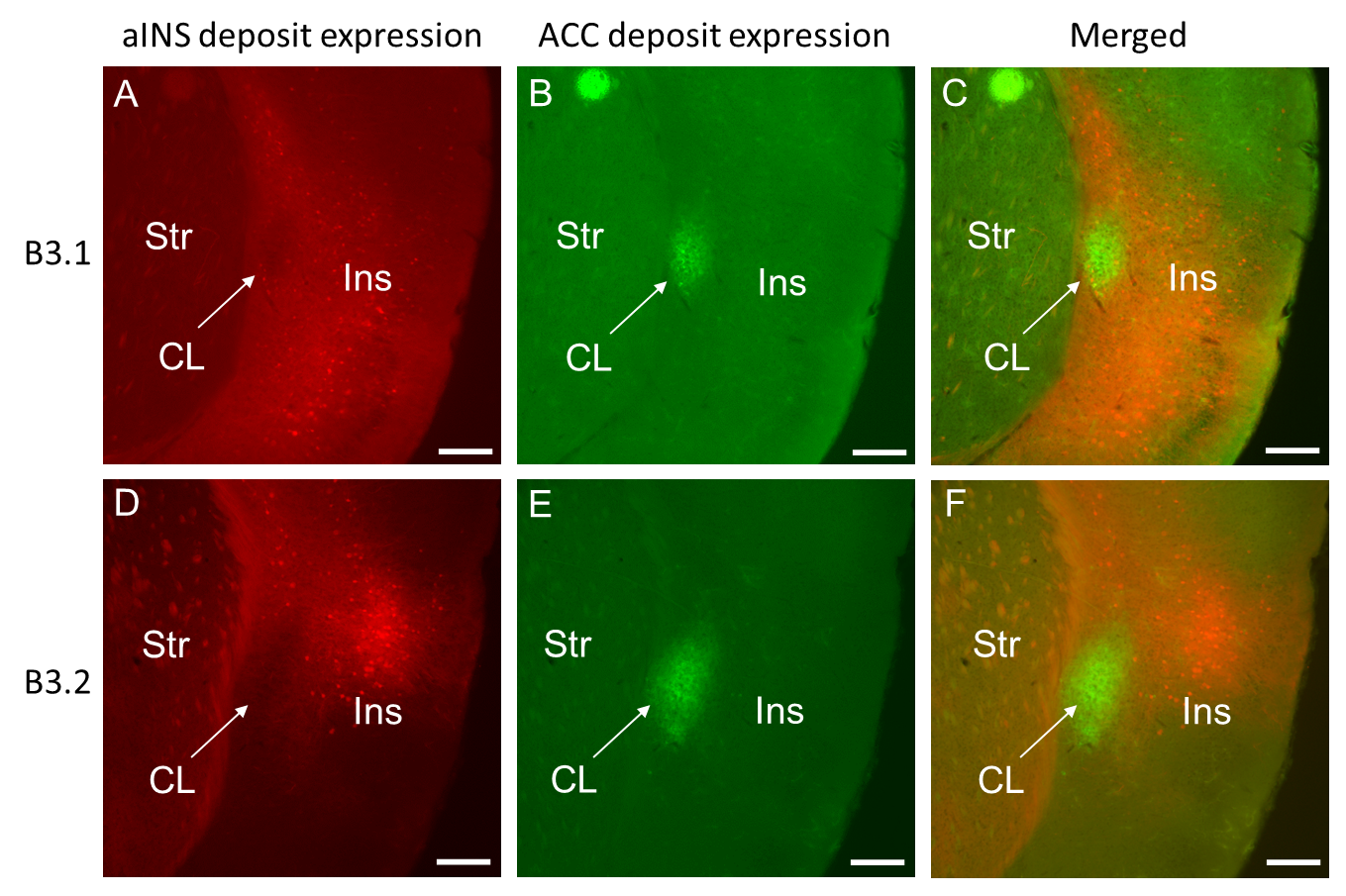

Supplement: Figure S2 — Photomicrographs of the rostral level of the claustrum and surrounding regions for case B3.1 (A–C) and B3.2 (D–F) (AP = +1.10 mm from bregma) showing retrograde and anterograde labeling following viral injections targeting aINS and ACC. Merge of panels (A) and (B) is shown in (C). Merge of (D) and (E) is shown in (F). Scale bars: 500 μm. Str, striatum; CL, claustrum; Ins, insular cortex. [file Image_2.TIF]

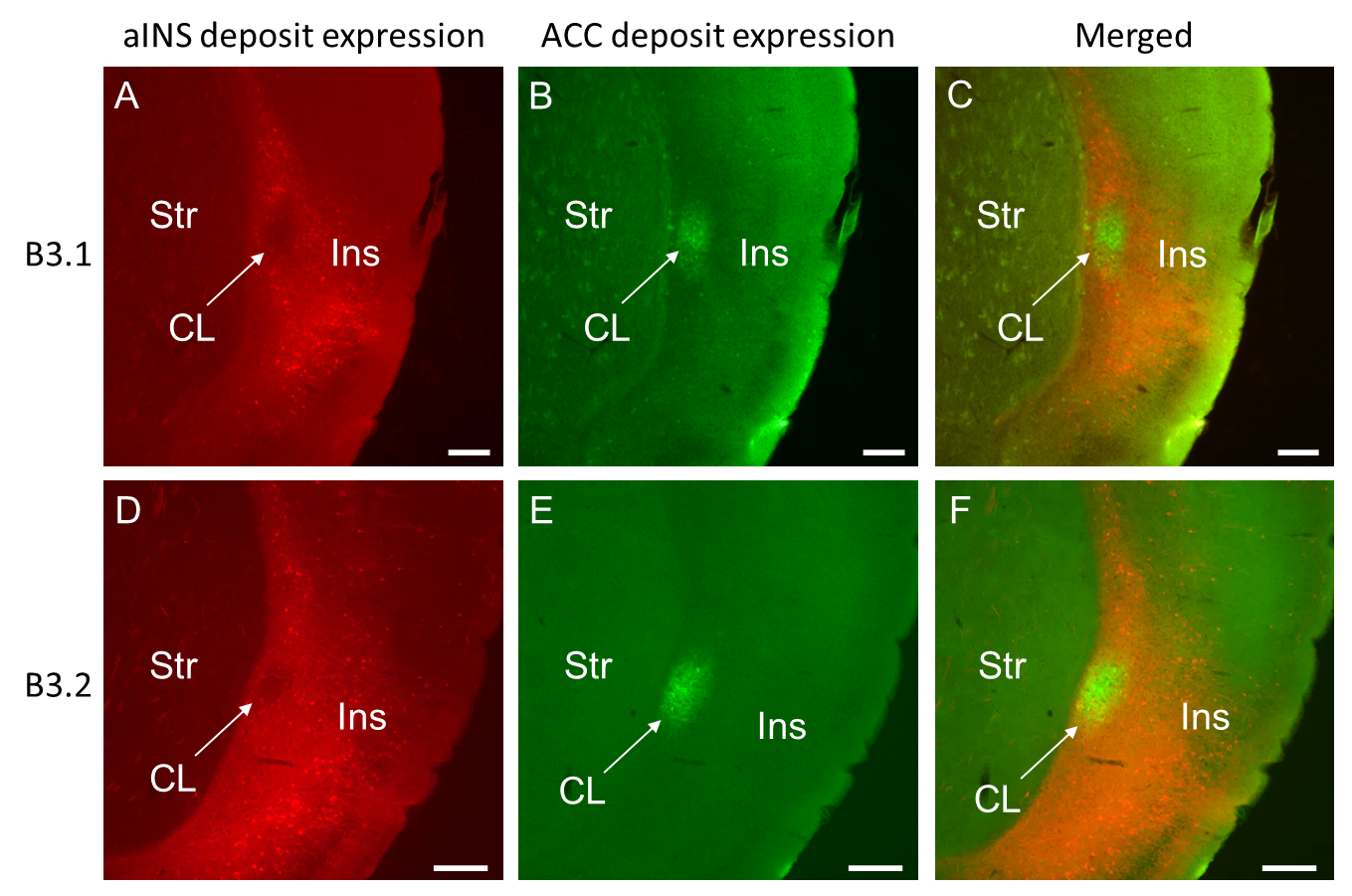

Supplement: Figure S3 — Photomicrographs of the mid level of the claustrum and surrounding regions for case B3.1 (A–C) and B3.2 (D–F) (AP = +0.50 mm from bregma) showing retrograde and anterograde labeling following viral injections targeting aINS and ACC. Merge of panels (A) and (B) is shown in (C). Merge of (D) and (E) is shown in (F). Scale bars: 500 μm. Str, striatum; CL, claustrum; Ins, insular cortex. [file Image_3.tif]

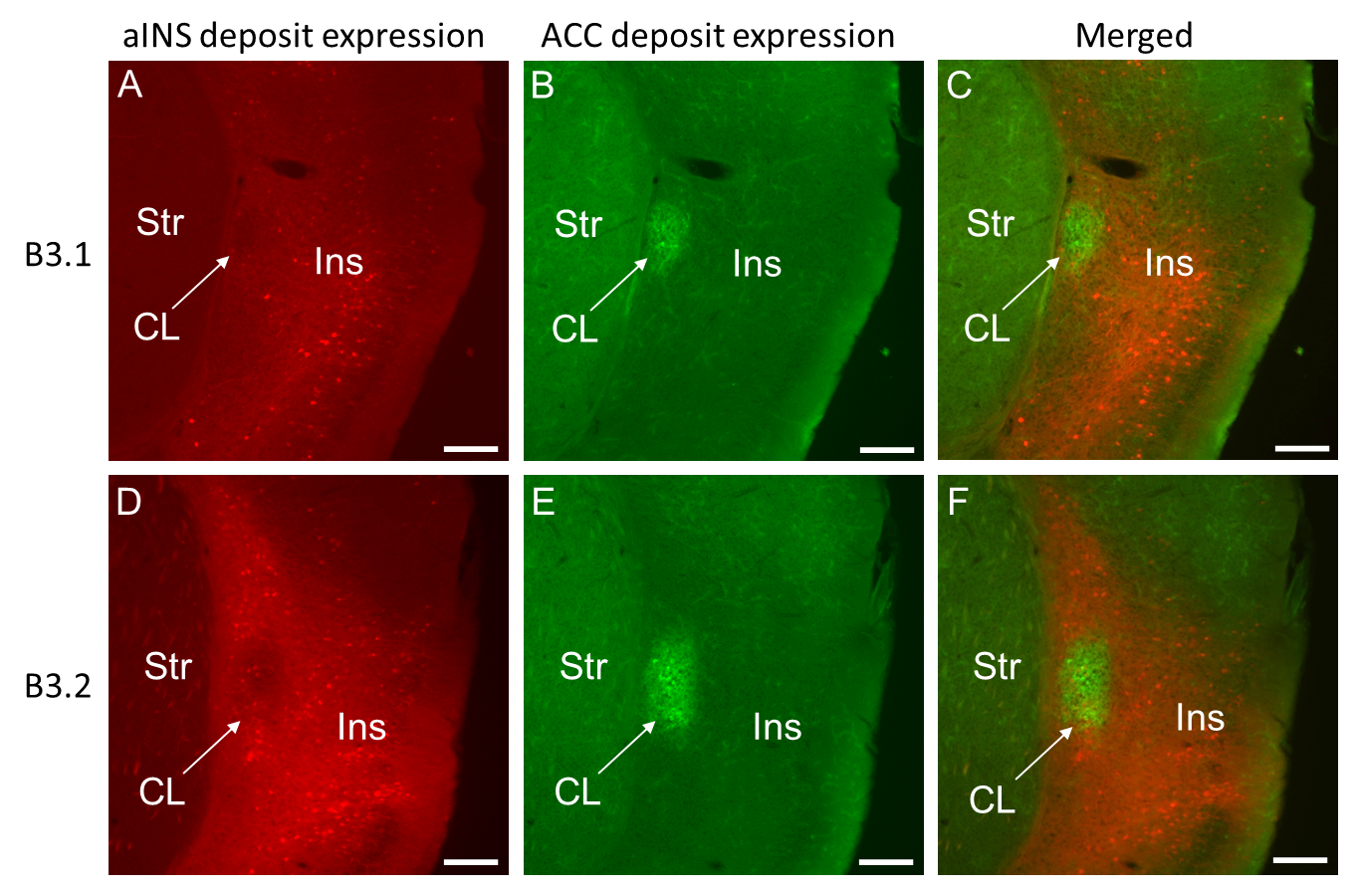

Supplement: Figure S4 — Photomicrographs of the caudal level of the claustrum and surrounding regions for case B3.1 (A–C) and B3.2 (D–F) (AP = +0.14 mm from bregma) showing retrograde and anterograde labeling following viral injections targeting aINS and ACC. Merge of panels (A) and (B) is shown in (C). Merge of (D) and (E) is shown in (F). Scale bars: 500 μm. Str, striatum; CL, claustrum; Ins, insular cortex. [file Image_4.TIF]
